# Vitamin E Phosphate Nucleoside Prodrugs: A Platform for Intracellular Delivery of Monophosphorylated Nucleosides

**DOI:** 10.3390/ph11010016

**Published:** 2018-02-06

**Authors:** Richard Daifuku, Michael Koratich, Murray Stackhouse

**Affiliations:** 1 Epigenetics Pharma, 9270 SE 36th Pl, Mercer Island, WA 98040, USA; 2 Southern Research, 2000 9th Avenue South, Birmingham, AL 35205, USA; mkoratich@southernresearch.org (M.K.); mstackhouse@southernresearch.org (M.S.)

**Keywords:** cancer, gemcitabine, nucleoside, nucleotide, prodrug, vitamin E, tocopherol, tocotrienol

## Abstract

Vitamin E phosphate (VEP) nucleoside prodrugs are designed to bypass two mechanisms of tumor resistance to therapeutic nucleosides: nucleoside transport and kinase downregulation. Certain isoforms of vitamin E (VE) have shown activity against solid and hematologic tumors and result in chemosensitization. Because gemcitabine is one of the most common chemotherapeutics for the treatment of cancer, it was used to demonstrate the constructs utility. Four different VE isoforms were conjugated with gemcitabine at the 5′ position. Two of these were δ-tocopherol-monophosphate (MP) gemcitabine (NUC050) and δ-tocotrienol-MP gemcitabine (NUC052). NUC050 was shown to be able to deliver gemcitabine-MP intracellularly by a nucleoside transport independent mechanism. Its half-life administered IV in mice was 3.9 h. In a mouse xenograft model of non-small cell lung cancer (NSCLC) NCI-H460, NUC050 at a dose of 40 mg/kg IV qwk × 4 resulted in significant inhibition to tumor growth on days 11–31 (*p* < 0.05) compared to saline control (SC). Median survival was 33 days (NUC050) vs. 25.5 days (SC) ((hazard ratio) HR = 0.24, *p* = 0.017). Further, NUC050 significantly inhibited tumor growth compared to historic data with gemcitabine at 135 mg/kg IV q5d × 3 on days 14–41 (*p* < 0.05). NUC052 was administered at a dose of 40 mg/kg IV qwk × 2 followed by 50 mg/kg qwk × 2. NUC052 resulted in inhibition to tumor growth on days 14–27 (*p* < 0.05) and median survival was 34 days (HR = 0.27, *p* = 0.033). NUC050 and NUC052 have been shown to be safe and effective in a mouse xenograft of NSCLC.

## 1. Introduction

Nucleosides can suffer from short half-lives as a result of deamination [1,2]. Also, when used for the treatment of cancer, they may not reach their intended target as a result of constitutive or acquired resistance, whether due to downregulation of nucleoside transport or kinases [3,4]. 

There have been a number of attempts to develop prodrugs of gemcitabine with modifications at the 5’ position of the sugar to address some of the shortcomings of its pharmacology and activity. Typically, these approaches have attempted to first and foremost prevent deamination by cytidine deaminase (CDA), the enzyme responsible for gemcitabine’s short half-life [5,6,7,8]. Less commonly, such approaches have attempted to bypass gemcitabine’s requirement for an intact nucleoside transport system for intracellular penetration [5,6,8] and deoxycytidine kinase (dCK) for phosphorylation to gemcitabine-monophosphate (MP) [7,8], the rate limiting step on the way to formation of the therapeutically active diphosphate (DP) and triphosphate (TP). Where such has been published, these constructs have tended to have a relatively short half-life [5,8]. 

Some isoforms of vitamin E (VE) have been shown to have activity against cancer, this is particularly true of δ-tocopherol and tocotrienols. Formal ranking of the relative biopotency of VE isoforms for suppression of cell growth and induction of cell death displays a reproducible relationship corresponding to δ-tocotrienol ≥ γ-tocotrienol > α-tocotrienol > δ-tocopherol >> γ and α-tocopherol [9]. As will be shown below, by linking a nucleoside to an appropriate vitamin E phosphate (VEP) isoform, it is possible to deliver to a cancerous cell a nucleoside-MP. Gemcitabine was selected as the model nucleoside because it is one of the most commonly used drugs in clinical oncology.

**Figure 1:**
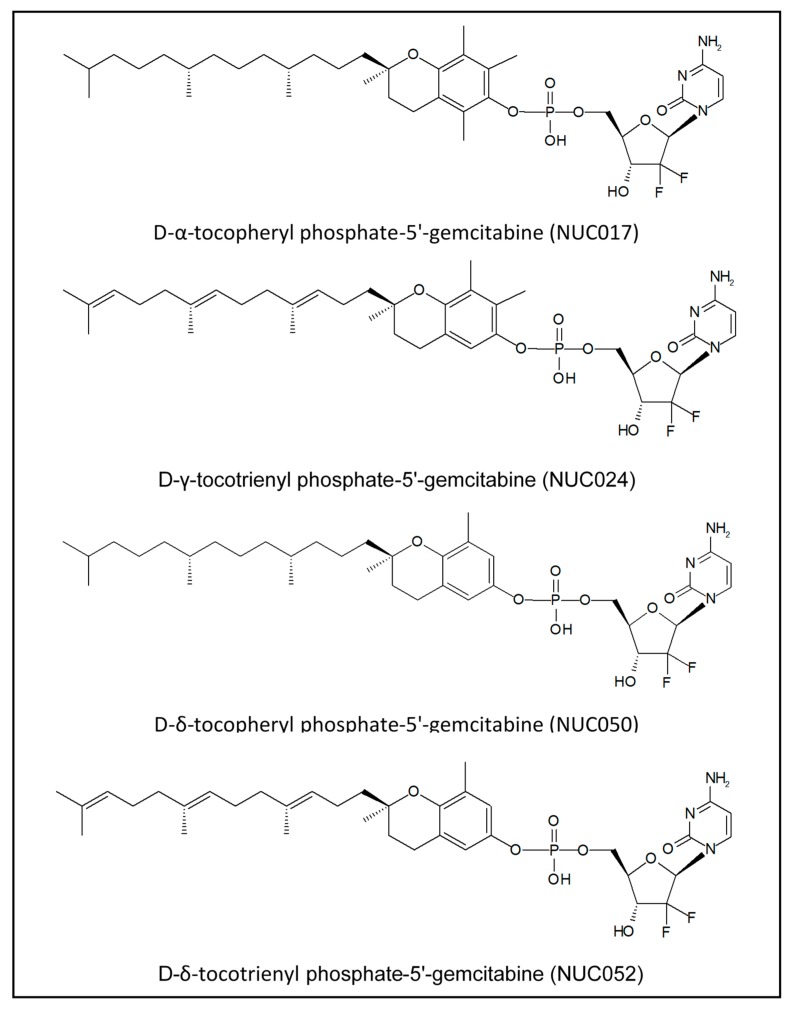
Isoforms of VE conjugated with gemcitabine phosphate.

Over the course of this research, four VEP isoforms of gemcitabine were synthesized (see Figure 1). 

## 2. Results

### 2.1. In Vitro Studies with VEP-Gemcitabine

Three of these VEP of gemcitabine, namely, NUC017, NUC024 and NUC050 were tested for in vitro activity and compared to gemcitabine (see Table 1).

All the VEP show similar activity but the VEP-gemcitabine do not. This is compatible with intracellular catabolism of the VEP-gemcitabine to VE and gemcitabine-MP. Because the most active of these three isoforms of VE is reported to be γ-tocotrienol, these results suggest that the activity of VEP-gemcitabine in vitro is related, at least in part, to the steric hindrance to enzymatic cleavage provided by methyl groups proximal to the VE-phosphate bond. Alpha isoforms have two methyl groups in close proximity to the bond, gamma one and delta none.

Subsequently, VEP gemcitabine prodrugs were tested in the presence and absence of dipyridamole (DP), an inhibitor of nucleoside transport and compared to gemcitabine. The GI_50_ of VEP-gemcitabine are relatively unaffected while those of gemcitabine increased by 18 to 80-fold (see Table 2). The difference in GI_50_ for gemcitabine in the MDA-MB-231 cell line in Table 1 and Table 2 DP(−), is most likely due to the biphasic growth pattern of that cell line, as these cells tend to increase proliferation the closer they get to confluence.

NUC050 was tested in vitro for activity against gemcitabine in wild type (WT) leukemic CEM cells and CEM cells deficient in dCK (dCK (−)). In the dCK (−) cells, gemcitabine is not phosphorylated to gemcitabine-MP, a precursor to the therapeutically active DP and TP. Gemcitabine GI_50_ went from 0.002 μM in dCK WT cells to 124.5 μM in dCK (−) cells, an increase of 62,250-fold. NUC050 GI_50_ went from 0.59 μM to 19.2 μM, an increase of only 32.5-fold, compatible with intracellular delivery of gemcitabine-MP (see Table 3).

### 2.2. Pharmacokinetics of NUC050

**Figure 2:**
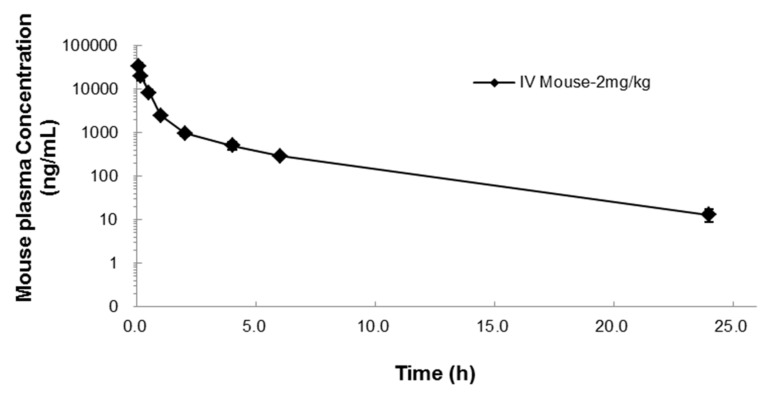
Mean concentration-time profile (± SD) after IV administration of 2 mg/kg NUC050 in mice.

The pharmacokinetics of NUC050 were evaluated in mice (see Figure 2 and Table 4).

### 2.3. Determination of NUC050 Maximum Tolerated Dose (MTD)

A dose of 277 mg/kg of NUC050 administered IV in saline resulted in the death of mice within minutes of injection and was felt to be compatible with surfactant like toxicity from the amphipathic nature of the prodrug. Hence, a nano-emulsion was developed and tested to attempt to mitigate such toxicity but did not change the MTD from 40 mg/kg qwk established in saline, based on limited testing. An MTD was not determined for NUC052.

### 2.4. Testing of NUC050 and NUC052 in a Xenograft of Human NSCLC

Activity of NUC050 was compared to that of NUC052 and gemcitabine in a mouse model of NSCLC (NCI-H460). In this study, the nano-emulsion formulation developed for NUC050 was compared to saline in animals treated with NUC050 and NUC052. Animals (*n* = 10/group) were initially treated with either (1) gemcitabine 120 mg/kg IP q3d, (2) NUC050 40 mg IV qwk (vehicle was saline (*n* =5) or nano-emulsion (*n* = 5)) or (3) NUC052 40 mg IV qwk (vehicle was saline (*n* = 5) or nano-emulsion (*n* = 5)).

Because of toxicity, the dose of gemcitabine was decreased to 80 mg/kg IP q3d after 4 doses. Likewise, changes were made to the vehicle for NUC050 and NUC052 after receipt of two doses. Indeed, after receipt of two doses of NUC050, it was noted that mice treated with NUC050 with saline as vehicle had better outcomes than those treated with nano-emulsion. The mean tumor volume was 183.4 mm^3^ vs. 513.0 mm^3^ (*p* = 0.031, student *t*-test, two-tailed) and the mean mouse weight 20.9 g vs. 18.3 g (*p* = 0.014, student *t*-test, two tailed). Consequently, the protocol was amended and saline was used as the vehicle. No vehicle toxicity or effect on efficacy was noted for NUC052, however, the mice receiving nano-emulsion were also switched to saline on the same study day and the dose increased to 50 mg/kg IV qwk.

**Figure 3:**
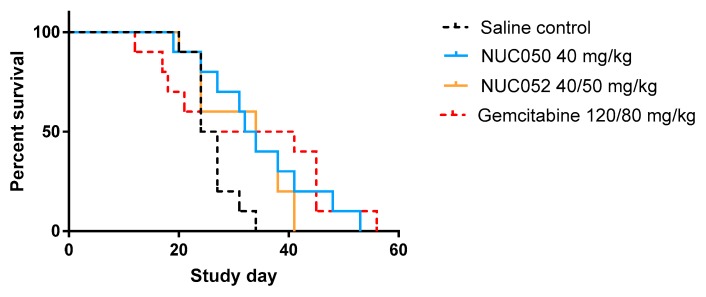
Survival proportions of NUC050, NUC052 and gemcitabine vs. saline control (SC) (*n* = 10 per group) in mice with human NSCLC NCI-H460 tumor implants. “Survival” refers to animals that were not removed from the study by death or per protocol euthanasia. Mice were administered the dose indicated IV qwk × 4 (study days 4–25) for NUC050. NUC052 was administered at 40 mg/kg IV qwk × 2 then 50 mg/kg IV × 2 (study days 4–25). Gemcitabine was administered IP at 120 mg/kg q3d × 4 and then 80 mg/kg IP q3d × 5 (study days 4–28). Median survival SC = 25.5 days. (1) NUC050 median survival = 33 days, hazard ratio (HR) vs. SC = 0.24 (*p* = 0.017, Log rank test). (2) NUC052 median survival = 34 days, HR = 0.27 (*p* = 0.033, Log rank test). (3) Gemcitabine median survival = 32.5 days, HR = 0.46 (*p* = 0.18, Log rank test).

In the NCI-H460 xenograft model of human NSCLC, treatment with NUC050 and NUC052 significantly improved mouse survival (*p* = 0.017 and *p* = 0.033, respectively), while treatment with gemcitabine at 120/80 mg/kg did not (*p* = 0.18) (see details in Figure 3).

**Figure 4:**
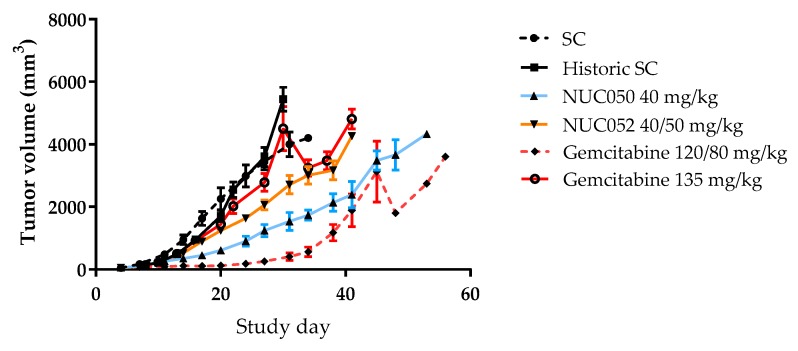
Comparison of mean tumor volumes (±SEM) in mice with human NSCLC. NCI-H460 implants treated with NUC050, NUC052 or gemcitabine (*n* =10 per group). SC mice are those treated contemporaneously with NUC050, NUC052 or gemcitabine 120/80 mg/kg as per regimen in Figure 3. Historic SC mice were from a previous experiment comparing SC to gemcitabine 135 mg/kg IV q5d × 3.

As shown in Figure 4, tumor volumes were significantly lower than SC (*p* < 0.05, student *t*-test, two tailed) for NUC050 on study days 14 through 31, with a nadir of −73%, while the same is true for NUC052 on study days 14 through 27 with a nadir of −45%. Tumor volumes were significantly lower on study days 14 through 41 for NUC050 vs. historic data for gemcitabine 135 mg/kg (*p* < 0.05, student *t*-test, two tailed). Tumor volumes were significantly lower (*p* < 0.05, student *t*-test, two tailed) for NUC050 compared to NUC052 on study days 17 through 34. The apparent drop in mean tumor volume after study day 45 with gemcitabine 120/80 mg/kg and study day 30 with gemcitabine 135 mg/kg is due to per protocol euthanasia of mice with tumors >4000 mm^3^, reflecting a greater heterogeneity in tumor response.

## 3. Discussion

There are two main isoforms of VE, tocopherols and tocotrienols. Most of the VE research to date has focused on α-tocopherol, though a growing body of literature supports a therapeutic role for tocotrienols. In part, the rationale for using VE as a prodrug carrier moiety is that some of the isoforms of tocopherols [10] and more so tocotrienols [9], have been reported to have anti-proliferative activity. Indeed, tocotrienols have shown activity against a number of different cancers, including breast [11,12], leukemia [13,14], liver [15], pancreas [16,17] and prostate [18], amongst others. In a study comparing the effects of tocopherols and tocotrienols on preneoplastic (CL-S1), neoplastic (-SA) and highly malignant (+SA) mouse mammary epithelial cell growth and viability in vitro, δ-tocopherol and δ-tocotrienol had the lowest GI_50_ on highly malignant +SA cells, with activity in the low μM range, and the highest GI_50_ in preneoplastic S1 cells [19].

In addition to well described antioxidative and pro-apoptotic functions, tocotrienols have demonstrated other interesting properties as possible treatments for cancer, including the induction of antitumor immunity, the inhibition of epithelial-to-mesenchymal transitions, the suppression of vascular endothelial growth factor tumor angiogenic pathway and more recently, chemosensitization and anti-cancer stem cell effects [20]. Chemosensitization was demonstrated in an orthotopic model of pancreatic cancer, where γ-tocotrienol was shown to inhibit pancreatic tumors and to sensitize such tumors to treatment by gemcitabine [16].

VEP-nucleoside prodrugs are designed to address two mechanisms of nucleoside resistance, namely in the case of gemcitabine, downregulation of nucleoside membrane transporters and dCK [21]. As is shown in the above data, NUC024 or NUC050 are unaffected by the presence of DP, in contradistinction to gemcitabine, suggesting that they bypass nucleoside membrane transporters. Likewise, NUC050 preserved most of its activity in CEM dCK (−) cells, suggesting that NUC050 delivered gemcitabine-MP intracellularly. The enzyme responsible for hydrolyzing NUC050 into δ-tocopherol and gemcitabine-MP has not been identified but could be a phosphatase or a phosphodiesterase. 

The half-life of gemcitabine is reported to be 0.28 h in mice [22], while NUC050 has a half-life of 3.9 h, a 13.9-fold increase. As gemcitabine’s primary mode of action, stalling of the DNA replication fork, is dependent on incorporation into DNA during S phase [23], a longer half-life should provide additional therapeutic benefit. As the half-life of gemcitabine is limited by deamination by CDA, these results suggest that NUC050 is not a substrate for CDA.

Unfortunately, gemcitabine demonstrated toxicity in the xenograft model of NSCLC, with one animal dying at dosing of 120 mg/kg and 6 more dying following a dose reduction to 80 mg/kg with the fifth dose onward. This despite a recent report where doses of 120 mg/kg q3d IP were administered in an NCI-H460 xenograft model without evidence of toxicity, albeit typically with a maximum of 4 doses. In this report, NCI-H460 tumors were noted as being insensitive to gemcitabine [24]. This illustrates the difficulty in administering safe and effective doses of gemcitabine in animal models or humans. In the clinic, levels of CDA can affect outcome of treatment with nucleosides that are substrates for CDA [25,26], furthermore mice have higher levels of CDA than humans [22]. Evidence of gemcitabine toxicity is confirmed by the lack of survival benefit. Comparing tumor growth in the presence of such toxicity is not meaningful, as tumors do not grow well in animals that are near moribund. However, the NCI-H460 xenograft model is robust and reproducible, as demonstrated by the overlapping tumor growth curves in both SC and historic SC groups. Thus, it is possible to compare data from a prior experiment with NCI-H460, where gemcitabine was administered at a dose of 135 mg/kg q5d × 3 without drug toxicity, which did not demonstrate evidence of tumor growth inhibition.

In the xenograft model of human NSCLC, NUC050 and its δ-tocotrienol variant, NUC052, demonstrated efficacy, with significant inhibition of tumor growth and significant improvement in survival compared to SC. Further, NUC050 demonstrated significant tumor growth inhibition compared to historic data with gemcitabine administered at a dose and regimen of 135 mg/kg q5d. In the case of NUC050, this was achieved while delivering a dose of only 14.5 mg of gemcitabine per week compared to a weekly dose of gemcitabine in the range of 280.0–186.7 mg/kg in animals administered 120 mg/kg reduced to 80 mg/kg q3d in the contemporaneous gemcitabine group, or 189 mg/kg per week in the 135 mg/kg q5d group. Thus, at weekly doses of gemcitabine equimolar to those administered with NUC050, gemcitabine would definitely not have shown antitumor activity. While tumor sizes were significantly lower in mice treated with NUC050 than NUC052, both demonstrated similar survival. It is possible that the use of lipid nano-emulsion vehicle adversely affected the results with NUC050 as following two doses of drug, mice that were treated with nano-emulsion had significantly lower weights and significantly larger tumor volumes than those treated with saline. As noted, such was not observed for NUC052. The toxicity of NUC050 observed at doses above its MTD is probably related to the amphipathic nature of the molecule and the possibility that it can act as a surfactant, which is compatible with the time course of death at the highest dose [27]. This can be considered the dose limiting toxicity (DLT) for NUC050. The lipid nano-emulsion had been specifically designed for NUC050 to attempt to decrease the risk of such toxicity and to increase tumor drug delivery through enhanced permeability and retention [28,29]. However, it should be noted that there were imbalances in the two groups upon treatment initiation. While differences in body weight were non-significant, tumor weights were larger in the group receiving nano-emulsion than the saline vehicle (mean tumor weight 63.0 mg vs. 44.4 mg, *p* = 0.04, student *t*-test, two tailed). Hence the adverse effect of this nano-emulsion on safety is better established than on efficacy and there were 3 animal deaths noted in the nano-emulsion group. It is possible that the nano-emulsion is preferentially taken up by the reticuloendothelial system, compromising mouse immunity and leading to some drug sequestration.

There are a number of gemcitabine prodrugs that have been proposed as a result of modification at the 5′ position of the sugar. CP-4126 consists of gemcitabine conjugated with elaidic acid esterified at the 5′ position. It is remarkable for a very short half-life, reported to be between 0.05 and 0.07 h following administration in dogs [5]. While showing initial promise, it failed in a Phase 3 clinical trial for the treatment of pancreatic cancer, as it did not show a significant difference in overall survival between patients treated with gemcitabine and those treated with CP-4126 [30]. More recently, a similar approach was taken, substituting a synthetic cardiolipin for elaidic acid and conjugating cardiolipin to gemcitabine by a succinate linker. This approach showed activity in vivo [6] but its development appears to have been abandoned. Another approach has been to synthesize a phosphoramidate prodrug of gemcitabine. The original construct was unable to bypass the nucleoside transport system but showed preservation of activity in dCK (−) cells [7]. A more recent development is an L-alanine gemcitabine phosphoramidate. It is reported to be less dependent on dCK and nucleoside transporters than gemcitabine, though it is also reported to have a short half-life in rodents because of circulating esterases [8]. It is currently in combination chemotherapy clinical trials for the treatment of ovarian cancer and biliary tract cancer [31].

The VEP-nucleoside platform differs from the approaches outlined above by bypassing the nucleoside transport system and delivering a nucleoside-MP intracellularly, while improving the pharmacokinetics of gemcitabine. In all cases, it differs by linking gemcitabine to an active carrier that has demonstrated chemosensitization and low toxicity. In animal models, at equimolar doses, NUC050 has demonstrated greater activity in a NSCLC model than NUC052, even though in vitro δ-tocotrienol demonstrates in the range of 10-fold more activity than δ-tocopherol [19]. The reasons for this are currently unknown but could be related to differences in cellular interactions with the phytyl tail, differences in pharmacokinetics or cellular penetration of the conjugate. It should be noted though that an MTD was not established for NUC052 and that such an MTD might be quite different from that of NUC050. It is possible that at a dose near its MTD, NUC052 would be more effective than was demonstrated herein. Relative scarcity of purified δ-tocotrienol and its sensitivity to oxidation has hampered its therapeutic development and required the comparison of NUC052 at doses near equimolar to those of NUC050, without the ability to perform pharmacokinetic or tolerability studies.

## 4. Materials and Methods

### 4.1. VEP-Gemcitabine Synthesis

NUC017, NUC024 and NUC050 were originally synthesized at Sonus Pharmaceuticals (Bothell, WA, USA). Subsequently, NUC050 and NUC052 were synthesized by NuChem Therapeutics (Montreal, QC, Canada).

### 4.2. Tumor Xenograft Studies

As per methods previously presented [32], the tolerability study and the tumor xenograft studies were performed at the same institution. These studies were carried out in strict accordance with the recommendations of the NIH Guide for the Care and Use of Laboratory Animals. The protocols were approved by the IACUC of Southern Research Institute (Birmingham, AL, USA) (AAALAC Accreditation: 000643). The animal protocol approval number is 15-03-009B from the Southern Research IACUC.

10^7^ tumor cells from culture in Matrigel™ of NCI-H460 human NSCLC were subcutaneously implanted in the flank of 1.75-fold the number of NCr-*nu/nu* mice required for the study. Study initiation began when the required number of mice had tumors of approximately 32 to 75 mm^3^ (target group mean tumor weight of approximately 50 mm^3^). In the historic controls, tumor volume at initiation of gemcitabine treatment was 100–180 mm^3^.

Mice with tumors in the proper volume range were arbitrarily assigned to groups. Mice received test article once a week for 4 weeks. Mice were observed daily for mortality and moribundity with weights and the tumor measurements taken twice weekly. Tumor volume was determined using the formula for an ellipsoid sphere: Length × Width^2^/2 = Volume (mm^3^). The experiments were scheduled to last for 60 days from the day of tumor implant. Any animal whose weight decreased more than 30% from the weight on the first day of treatment or whose tumor reached 4000 mm^3^ in volume, ulcerated or sloughed off, or was moribund was euthanized prior to study termination.

### 4.3. In Vitro Activity

As per methods previously presented [32], cells were grown in appropriate medium for the cell line of interest. 96 well plates of each cell line were seeded with 5000 cells per well and left overnight. Drug exposed cells were incubated at 37 °C for 72 h. At the end of the 72-h exposure period, plates were removed for the CellTiter-Glo^®^ assay. Luminescence was recorded on a Synergy 4.0. Assays were performed in triplicate.

### 4.4. Pharmacokinetic Study

This study was carried out in strict accordance with the recommendations of the NIH Guide for the Care and Use of Laboratory Animals. The protocol was approved by the Institutional Animal Care and Use Committee (IACUC) of Eurofin Panlabs Taiwan Ltd. (Taipei, Taiwan) (AAALAC Accreditation: 001553). 

As per methods previously presented [32], a plasma calibration curve was generated and a reportable linear range determined, along with the lower limit of quantitation. Twenty-four mice were administered 2 mg/kg NUC050 IV, *n* = 3 animals per time point at 8 time points (3, 10, 30, 60, 120, 240, 360 and 1440 min) and control animals (*n* = 3, for drug-free blood collection). Blood aliquots (300–400 µL) were collected via cardiac puncture from anesthetized mice in anticoagulant coated tubes, then kept on ice and centrifuged at 2500× *g* for 15 min at 4 °C. The plasma was harvested and kept frozen at −80 °C. Plasma samples were processed using acetonitrile precipitation and analyzed by HPLC-MS/MS.

### 4.5. Nano-Emulsion

The nano-emulsion (Latitude Pharmaceuticals, San Diego, CA, USA) contains an oil phase consisting of an injectable oil and lecithin and an aqueous phase comprising a tonicity adjuster, a stabilizer and water. The emulsions are of oil-in-water type with the mean oil droplets less than 100 nm. The vehicle is at neutral pH (5–7) and about isotonic. 

## 5. Conclusions

Using VEP-gemcitabine as a model, the above research has shown that linking a VEP to a nucleoside results in the intracellular delivery of a nucleoside-MP. VEP-gemcitabine has also demonstrated a desirable pharmacokinetic profile and in particular a prolonged half-life. The approach of linking an appropriate VE isoform to a therapeutic nucleoside provides the benefit of chemosensitization and can be used to overcome tumor resistance or to deliver to cells therapeutic nucleosides that are poorly phosphorylated because of their design. Further formulation development is necessary to overcome the dose limiting toxicity associated with these constructs and increase the safety and efficacy of VEP-nucleosides.

## Figures and Tables

**Table 1:** Comparison of in vitro GI_50_ between gemcitabine, VEP isoforms and VEP gemcitabine prodrugs NUC017, NUC050 and NUC024.

	Cancer Cell Line		
Compound	Breast MDA-MB-231 (μM)	Non-Small Cell Lung NCI-H460 (μM)	Colon HCT-116 (μM)
Gemcitabine	0.11	0.02	0.01
α-tocopheryl phosphate	23.40	52.24	48.86
NUC017	22.70	23.75	26.13
δ-tocopheryl phosphate	29.56	69.67	70.58
NUC050	5.08	1.69	3.67
γ-tocotrienyl phosphate	26.42	69.14	55.71
NUC024	4.90	4.75	4.01

**Table 2:** Comparison of GI_50_ of gemcitabine, NUC050 and NUC024 in the presence or absence of dipyridamole (DP).

	GI_50_ (μM)					
	Breast MDA-MB-231		Non-Small Cell Lung NCI-H460		Colon HCT116	
Compound	DP (−)	DP (20 μM)	DP (−)	DP (20 μM)	DP (−)	DP (20 μM)
Gemcitabine	3.08	56.77	0.02	0.82	0.03	2.39
NUC050	17.16	23.30	2.14	1.47	3.07	6.74
NUC024	30.34	27.77	7.16	15.98	5.55	12.61

**Table 3:** Comparison of GI_50_ of gemcitabine and NUC050 in CEM WT cells and cells deficient in dCK.

	GI_50_ (μM)	
Cell Line	Gemcitabine	NUC050
CEM WT	0.002	0.59
CEM dCK (−)	124.5	19.2

**Table 4:** Pharmacokinetic parameters after 2 mg/kg IV NUC050 administration in mice. Where, T_1/2_: half-life, C_0_: concentration at time 0, AUClast: area under the curve to the last time point, AUCinf: area under the curve extrapolated to infinity, AUC Ext: Proportion of AUC extrapolated, Vss: volume of distribution at a steady state, CL: clearance, MRT: mean residence time.

T_1/2_ (h)	C_0_ (ng/mL)	AUClast (h·ng/mL)	AUCinf (h·ng/mL)	AUC Ext (%)	Vss (L/kg)	CL (mL/min/kg)	MRT (h)
3.9	42,351	19,028	19,101	0.38	0.2	0.8	1.8
